# Intensive care admission triage for a pandemic: are government tools acceptable to UK intensivists?

**DOI:** 10.1186/cc9886

**Published:** 2011-03-11

**Authors:** DT Ashton-Cleary, NV Freeman, A Tillyard

**Affiliations:** 1 Derriford Hospital, Plymouth, UK; 2 Torbay Hospital, Torbay, UK

## Introduction

Triage criteria recommended by various governmental bodies are part of a process to cope with increased demand for intensive care resources during a pandemic [1]. It is unknown whether UK intensive care physicians agree with the proposed criteria that could automatically exclude a patient from receiving ICU care if adopted.

## Methods

We conducted an online survey amongst the members of the UK Intensive Care Society. We asked respondents to grade their opinion about each criterion of a Department of Health (DoH) triage tool and provide some additional information about their own health. We used Cronbach's alpha (CA) to assess how close the opinions of the respondents were with regard to each criterion and each of three sets of criteria. We used a chi-squared analysis to see whether these factors differed between intensive care consultants and nonconsultants.

## Results

A total of 550 questionnaires were returned; 182 (33.1%) were from intensive care consultants. For six of the DoH 11 criteria, the agreement score was >4/5 indicating agreement or strong agreement. For both consultants and nonconsultants, the CA was >0.8 (significant inter-responder agreement). A total 19.4% of those currently meeting exclusion criteria and 34.6% of those in good health would give up the chance of a level 3 bed voluntarily if they fulfilled one of the proposed criteria during a pandemic.

## Conclusions

The results indicate a general acceptance of the requirement for triage but nearly 40% have significant reservations about the proposed tool. Sixty-five to 80% of respondents would not withdraw from the triage process in a pandemic even if they knew the proposed criteria would exclude them. While approximately 60% of respondents accepted the triage tool, it seems the majority would not wish it to be used to determine their own care.
